# Applying the RE-AIM conceptual framework for the promotion of physical activity in low- and middle-income countries

**DOI:** 10.1590/1518-8345.1894.2923

**Published:** 2017-09-18

**Authors:** Rebecca E. Lee, Karla I. Galavíz, Erica G. Soltero, Jose Rosales Chavez, Edtna Jauregui, Lucie Lévesque, Luis Ortiz Hernández, Juan Lopez y Taylor, Paul A. Estabrooks

**Affiliations:** 1 PhD, Professor, College of Nursing and Health Innovation, Arizona State University, Phoenix, AZ, United States.; 2 Post-doctoral fellow, Center for Health Promotion and Disease Prevention, Arizona State University, Phoenix, AZ, United States.; 3 PhD, Researcher, Texas Obesity Research Center, University of Houston, Houston, TX, United States.; 4 Researcher, School of Human Evolution and Social Change, Arizona State University, Tempe, AZ, United States.; 5 PhD, Coordinator, Departamento Medicina Preventiva, Secretaria de Salud, Guadalajara, JA, Mexico. Full Professor, Centro Universitario de Ciencias de la Salud, Universidad de Guadalajara, Guadalajara, JA, Mexico.; 6 PhD, Professor, School of Kinesiology & Health Studies, Queen’s University, Kingston, ON, Canada.; 7 PhD, Professor, Departamento de Atención a la Salud, Universidad Autónoma Metropolitana unidad Xochimilco, Ciudad de México, CX, Mexico.; 8 MD, MSc, Director, Instituto de Ciencias Aplicadas a la Actividad Física y Deporte, Centro Universitario de Ciencias de la Salud, Universidad de Guadalajara, Guadalajara, JA, Mexico.; 9 PhD, Adjunct Professor, Family and Community Medicine, Virginia Tech Carilion School of Medicine, Roanoke, VA, United States.

**Keywords:** Latin America, Strategies, Evaluation of Programs, Exercise, Health Plan Implementation

## Abstract

**Objective:**

the RE-AIM framework has been widely used to evaluate internal and external validity of interventions aimed to promote physical activity, helping to provide comprehensive evaluation of the reach, efficacy, adoption, implementation and maintenance of research and programming. Despite this progress, the RE-AIM framework has not been used widely in Latin America. The purpose of this manuscript is to describe the RE-AIM framework, the process and materials developed for a one-day workshop in Guadalajara, and the acceptability and satisfaction of participants that attended the workshop.

**Methods:**

lecture, interactive examples and an agenda were developed for a one-day RE-AIM workshop over a three month period.

**Results:**

thirty two health care practitioners (M age = 30.6, SD=9.9 years) attended the workshop. Most (100%) rated the workshop as credible, useful (100%) and intended to apply it in current or future research (95%).

**Conclusion:**

results suggest intuitive appeal of the RE-AIM framework, and provide a strategy for introducing the utility and practical application of the framework in practice settings in Mexico and Latin America.

## Introduction

Regularly performed physical activity (PA) is an international health priority^(1, 2)^. Physical inactivity is endemic in Mexico, and a majority of Mexican children (58.6 %) and many adults (19.4%) fail to meet physical activity recommendations^3^ ^-^ ^6^. Like other low and middle income countries (LMIC), the Mexican population is at high risk for developing health compromising conditions related to physical inactivity^7^ ^-^ ^9^. Implementing research and practice in LMICs provides an opportunity to understand and investigate the application of research techniques such as the RE-AIM framework, that have, to date, almost entirely focused on high income countries. 

The RE-AIM framework has been broadly applied in the US as well as other high -income countries across a wide array of PA-related research and programming; however, use of the framework has not been established in LMICs. The RE-AIM framework provides a model to inform the design, implementation and evaluation of physical activity, so its introduction in a LMIC country, like Mexico, is timely and promising. RE-AIM components of reach, efficacy/effectiveness, adoption, implementation and maintenance (individual and organizational) have been used to review internal and external validity of PA interventions using behavior change theories, school-based strategies, telephone-delivered strategies, workplace interventions, and interventions targeting cancer survivors^10^ ^-^ ^14^. 

Similar to other LMIC, infrastructure and public health needs in Mexico have historically focused on the prevention and treatment of infectious diseases. More recently, behavioral research has started to focus on preventing chronic conditions. In the case of Mexico, national statistics describing high rates of obesity and type 2 diabetes have led to recent sweeping policy changes. These commenced in 2012 with a change in political leadership, along with international supports, leading to activities throughout Mexico to improve cardiometabolic health, with a focus on increasing PA among all Mexicans^2^. The Mexican health statistics and policy changes have provided a favorable macro-level context to plan and evaluate current research and evidence-based interventions to increase PA in Mexico. The use of frameworks like RE-AIM allow for the possibility of achieving a public health impact by focusing on a range of outcomes such as the reach, efficacy, adoption, implementation and maintenance of these strategies.

Like many LMIC, health promotion efforts in Mexico tend to rely on both clinic-based and community public health programming^15^ ^-^ ^19^. Programs that have a broad reach into the population while demonstrating robust effectiveness across subgroups within the population can have a strong public health impact and may be considered for broad dissemination to other communities, systems and regions^20^ ^-^ ^21^. Despite their promise, a review of PA public health programs in Mexico showed that programs might report reach and adoption, but there was poor monitoring and evaluation of factors related to effectiveness, implementation and maintenance^22^. The evidence supporting these programs is insufficient for determining public health impact, limiting the ability to implement these programs on a broader state or national scale^9^ ^,^ ^15^ ^,^ ^19^ ^,^ ^23^. Current methods of evaluation and reporting exclude key areas that would facilitate dissemination about the expertise of those delivering the program, the program components, implementation activities and costs, the long-term sustainability of the programs and health and behavior outcomes for participants^16^. 

Despite the ability of the RE-AIM framework to help researchers and practitioners evaluate and assess the public health impact, there has been little use of the framework in Latin American countries such as Mexico, in part driven by a lack of knowledge and expertise. There is a strong need to develop capacity among public health practitioners and health promoters so that efforts can be systematically evaluated in order to disseminate successful programs across Mexico and to other LMICs with sizeable Hispanic populations. In this manuscript, we present the development and outcomes of a RE-AIM training workshop delivered in Guadalajara, Mexico including examples of planning and evaluation across the RE-AIM framework. 

## Method

The work described herein is the result of nearly a decade of multinational collaboration that developed through a participatory process involving researchers from Canada, the United States and Mexico. The primary goals of the partnership have been to increase scientific capacity and infrastructure in México with the express objective of discovering, enhancing and implementing strategies across multiple settings to increase PA among Hispanics or Latinos throughout North America. The workshop was conceived as a strategy to meet both goals by improving the quality of evaluation of public health programming for PA in Mexico using culturally relevant and interactive examples and was presented as a pre-congress session to the *Congreso Internacional de Avances en Medicina de “Hospitales Civiles de Guadalajara”* in 2014.

### The RE-AIM Framework

RE-AIM is comprised of five indicators: Reach, Efficacy/Effectiveness, Adoption, Implementation and Maintenance^24^. These indicators can be used in the evaluation of programs, procedures, policies or scientific studies. Reach is defined as the number or percentage of the population and the representativeness of those included in the program or study. Efficacy and effectiveness measure change in the variable of interest as well as impact on quality of life and adverse outcomes. Adoption measures the number, percentage, and representativeness of staff and settings involved. Implementation assesses the extent to which a program or policy is delivered consistently, and the time and costs of the program. Maintenance assesses the long-term effects and attrition in the project, both of individuals and organizations. This includes the extent of discontinuation, modification, or sustainability of program. 

Although there are other strategies for measuring process factors related to implementation of interventions that can describe internal and external validity, the RE-AIM framework has the advantages of being contextual, practical and having robust evidence of its applicability across a wide array of interventions, populations, settings and health behaviors. RE-AIM offers a systematic framework for expanding beyond the usual measures of efficacy and effectiveness, to the broader criteria of internal and external validity. RE-AIM moves away from a paradigm that focuses on the magnitude of effect as a key indicator for program/intervention impact towards a broader conceptualization of public health impact that includes reach, organizational adoption, and sustainability. RE-AIM attends to the characteristics of programs and interventions that ensure these can be readily adopted, widely implemented, and sustained. RE-AIM has been used to plan health interventions, evaluate health interventions, evaluate health policy impact, assess the literature, and to compute composite metrics to estimate intervention impact^25^ ^-^ ^29^. 

### Participants

Thirty two health practitioners (*M* age = 30.6, SD=9.9 years) participated in an eight hour workshop in Guadalajara. Participants represented a broad array of health professions including, PA trainers (28.1%, *N*=9), physicians (28.1%, *N*=9), teachers (15.6%, *N*=5), nutritionists (9.4%, *N*=3), nurses (6.3%, *N*=2), community workers (6.3%, *N*=2), a psychologist (3.1%, *N*=1) and one student (3.1%, *N*=1). Cost of attendance was included as part of the overall Congress fees, and attendees were able to apply for continuing education credits by virtue of their participation. 

### Measures

Before the workshop commenced, all participants completed items on an anonymous, pre-workshop, simple paper and pencil survey and indicated their age and health professions. Participants also indicated whether they had heard of RE-AIM before, and whether they evaluated their PA programming in their work settings, and how they hoped to use the skills that they gained in the workshop. 

Post-workshop, participants completed the remaining items on the survey. They were asked to rate the amount of new information that they learned in the workshop on a scale of 1 (learned no new information) to 7 (a lot of new information), how credible they found the information on a scale of 1 (not credible) to 7 (very credible). Participants also rated how likely it was that they would use the information gained either in their current profession or in the next six months on a scale of 1 (not very likely) to 7 (very likely). Last, participants indicated how interested they were in learning more about the issues presented in the workshop in a short course in the future on a scale of 1 (not interested) to 7 (very interested).

Surveys were distributed by the team, and later returned in a single file folder. Both surveys were developed for use with this workshop.

### Development of the workshop

#### Classroom style lecture

**Figure 1:**
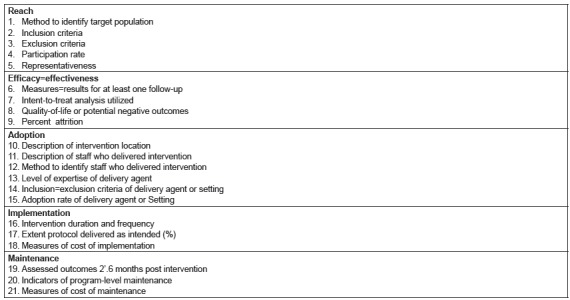
RE-AIM components used in the development and implementation of the workshop.

The first half of the workshop (~4 hours) included classroom style lecture, where one of the co-authors provided information and examples of the RE-AIM framework using PowerPoint slides. This initial approach was based on information from previous literature reviews suggesting little awareness of the RE-AIM framework in México and from key information gleaned from the Mexican members of the multinational collaboration (described above). First, well-known examples from studies done in Latin American countries were presented to show that, although efficacy or effectiveness are widely reported in the published literature, information was insufficient to determine which interventions worked, for whom and under which conditions. For example, participants were polled during the lecture to consider examples which illustrated how to report factors related to scalability, such as cost. Another example was how retention and sustainability might make a difference in which program was more likely to be adopted across organizations. Polls were based on items from the RE-AIM measure presented in Figure 1, previously developed by Glasgow et al. and later expanded by Allen et al.^28^ ^-^ ^29^. 

#### Interactive examples

Materials were developed over a two month period involving three teleconference calls among the authors. Each example was developed by a team involving one Mexican partner and one Canadian/US partner. Examples were then reviewed by the group, and inconsistencies or quandaries were discussed and resolved via teleconference. Materials were developed to have plausibility within a Mexican context and were translated and back translated to Spanish by bilingual native Mexican Spanish speakers. Examples were developed around three general content areas: Policy and environmental changes, prevention and public health, self- management of chronic diseases. Subgroups discussed each content example independently and then regrouped as a large group to talk through the examples together. 

## Results

### Workshop Agenda and Materials 

**Figure 2:**
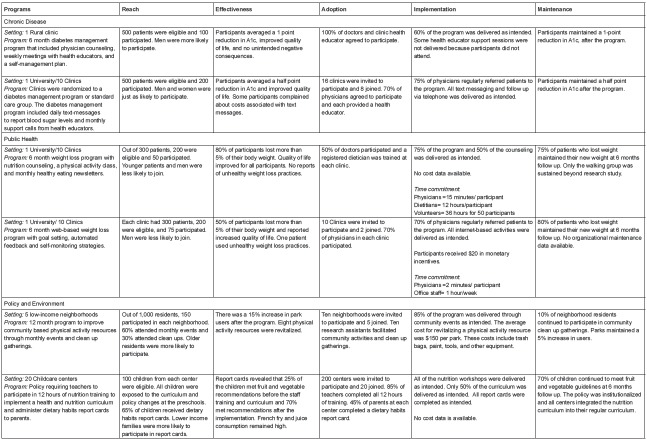
Program examples used for interactive activities to demonstrate RE-AIM constructs

**Figure 3:**
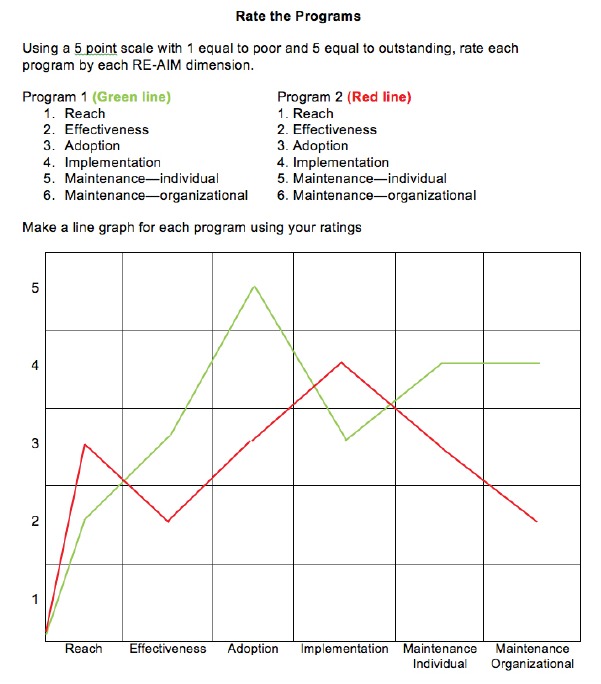
Sample of a completed rating form to compare programs in Activity 1.

The resulting workshop agenda featured classroom style learning for the entire group in the first half of the session, followed by a small group, interactive activities. After the small groups exercise, the entire group reconvened to discuss the activities and answer questions. Activity 1 presented two programs with information about the reach, efficacy/effectiveness, adoption, implementation and maintenance of each example. A summary is presented in Figure 2. Participants were asked to review the examples and then rate the two programs on a single grid, using a five-point rating system, where 1 equaled poor and 5 equaled excellent. As presented in Figure 3, this provided a way to visually compare each of the programs and evaluate which might be a better fit to meet the organizations goals. 

In Activity 2, participants were asked to describe their own intervention idea, indicate which RE-AIM dimensions were targeted for intervention, and which might be described, but would not be targeted for intervention. Participants then had the opportunity to describe challenges that they would face in their organization.

### Participant outcomes

Thirty two researchers and practitioners attended the RE-AIM training. Before the workshop, only five (15.6%) attendees had previously heard of or used the RE-AIM framework. About one fourth (*N*=8, 25%) used program evaluation tools in their work. Twenty-three (72%) indicated that they hoped to use the framework to guide their work in evaluating current and planning future projects, two (6%) in future academic training (e.g., thesis), and seven (22%) did not respond to the question of how they hoped to use the skills gained in the workshop.

Twenty-one participants completed the post workshop survey. After attending the training, 85.7% (*N*=18, *M*=6.33, SD=0.97) ranked a 6 or 7 indicating that they had learned a lot of new information in the workshop, and all ranked the information gleaned as credible (100%, *N*=21, *M*=6.71, SD=.46). Nearly all rated a 6 or 7 indicating that they would likely use the information acquired in the workshop in the current position (95%, *N*=20, *M*=6.71, SD=.56) or hope to use it in the next six months (95%, *N*=20, *M*=6.62, SD=.60). All participants indicated that they would be interested in participating in a short course to learn more about the RE-AIM framework (100%, *N*=21, *M*=6.85, SD=.36).

## Discussion 

This manuscript describes the development and reactions to the first RE-AIM workshop for public health practitioners delivered in Mexico. Relying on empirical evidence, we carefully constructed a culturally relevant workshop that produced favorable knowledge acquisition among a group of public health practitioners in Mexico. It was clear from the response to the workshop that there was intuitive appeal for RE-AIM to this audience of practitioners who rated the information presented as useful and credible, and desired to learn more about it for current or future use in their work. 

In a time where PA programming has reached the public health agenda, opportunities for improving current programs, disseminating successful strategies and informing future public health initiatives are numerous. RE-AIM can be used for evaluating the reach, impact and implementation of current PA initiatives at the participant, organization and policy levels. Further, by addressing cost, adoption and implementation factors, RE-AIM can guide the expansion and sustainability of successful programs across Mexico. By focusing on factors associated with the reach, real-world implementation, dissemination, and sustainability of successful PA programs, such initiatives could reach broader populations, a wide range of organizations and inform decision makers. 

Public health programming in Mexico is to some extent driven by political priorities; thus, when administrations change, so do public health promotion priorities. For example, in 2012, the change in administrations and health promotion strategy emphasized greater promotion of health behaviors related to obesity and diabetes in Mexico. This laudable change in priorities had effects throughout the country, increasing policies and programming at the national and state level that focused on PA and nutrition. Rapid shifts in priorities on a national level may leave little time to plan careful evaluation, even in the case of very positive changes as have been seen in Mexico. In the context of a politically driven public health system, individual practitioners, although motivated and well trained, may have little control over planning, implementation and evaluation of programming. RE-AIM framework can be helpful to show how these political priorities put emphasis on some aspects of programs such as reach, while other dimension are neglected including implementation or maintenance. During the workshop, participants made additional system considerations, concerning the environment and specific policies for Mexico, and focused on how to apply the RE-AIM framework in such contexts and political conditions.

Strengths of the reported experience included the dissemination of a well-researched and validated evaluation framework, careful crafting of relevant examples and interactive exercises, development and delivery by an experienced, multilingual team of researchers and practitioners and a very positive reception from a group of inexperienced participants. This study relied on a relatively small sample size, insufficient for more elaborate statistical modeling. Although pre- and post-workshop surveys were completed anonymously, self-report measures can suffer from response bias. Future research and development in this area should emphasize continued development of locally relevant examples and interactive activities and streamlining of RE-AIM measures to help aid adoption on a broader scale. From a translational science perspective, public health programming evaluation derived from the RE-AIM framework can help practitioners and policy makers predict the behavior of organizations and key stakeholders who are instrumental in the wide-spread adoption and successful implementation of evidence-based programs. Understanding where programming is both successful and challenging in the process of adoption and implementation can in turn drive their potential for sustainability, needed adaption for scaling up, and areas ripe for expansion. 

Despite the strengths and potential for gain that the RE-AIM framework offers, there are areas of additional development and future research, particularly in Mexico. For example, most attendees had a clear and immediate understanding of efficacy/effectiveness, but struggled with some of the other concepts such as adoption. It was very helpful to have carefully constructed and clearly translated definitions and examples for exercises and discussion to help illustrate how the RE-AIM could be applied in the local context. Examples based on real life programs would have helped to anchor constructs even more clearly; however, we struggled to find programs from Mexico that reported enough RE-AIM indicators to use as examples^22^. It was a great strength to have an experienced team of presenters that included native speakers to help explain constructs to the diverse audience and overcome barriers to understanding. Last, the RE-AIM framework may help enhance teamwork by providing clear definitions and real-world understanding of health outcomes. Workshops such as this one can promote understanding, communication, and planning across multiple disciplines, enhancing interprofessionalism and successful teamwork^30^. 

## Conclusion

Although the RE-AIM framework was initially conceptualized as a model to evaluate research, in reality, most interventions are tempered by the community in which they are administered. Programs can operationalize processes that involve local organizations and the intervention community itself. For example, in health care settings, interventions and programming must be designed to integrate within the existing organizational processes as well as the abilities of the practitioners and the reach of the clinic. There is an important role for partnerships between practitioners and researchers so that practice realities can inform research ideals. There is also room for simplification and streamlining of measures of the RE-AIM constructs for use under real-world conditions with limited resources. Next steps for translation of the RE-AIM framework include adapting the RE-AIM strategy to evaluate programming that builds on existing resources, requires little advance planning, and that is reasonably easy to accomplish given existing resources. 
